# Diagnostik und Therapie der Riesenzellarteriitis

**DOI:** 10.1007/s00115-021-01216-8

**Published:** 2021-11-03

**Authors:** Markus Kraemer, Jana Becker, Thorsten Alexander Bley, Andreas Steinbrecher, Jens Minnerup, Bernhard Hellmich

**Affiliations:** 1 Klinik für Neurologie, Alfried Krupp Krankenhaus Rüttenscheid, Alfried-Krupp-Straße 21, 45130 Essen, Deutschland; 2 Klinik für Neurologie, Medizinische Fakultät, Heinrich Heine Universität Düsseldorf, Düsseldorf, Deutschland; 3 Klinik für Neurologie und klinische Neurophysiologie Philippusstift, Essen, Deutschland; 4 Institut für Diagnostische und Interventionelle Radiologie, Universitätsklinikum Würzburg, Würzburg, Deutschland; 5 Klinik für Neurologie, Helios Klinikum Erfurt, Erfurt, Deutschland; 6 Klinik für Neurologie mit Institut für Translationale Neurologie, Universitätsklinikum Münster, Münster, Deutschland; 7 Klinik für Innere Medizin, Rheumatologie und Immunologie, Medius-Klinik Kirchheim unter Teck, Kirchheim unter Teck, Deutschland

**Keywords:** Riesenzellarteriitis, Diagnose, Therapie, Glukokortikoide, Glukokortikoideinsparende Therapie, Tocilizumab, Methotrexat, Giant cell arteritis, Diagnosis, Therapy, Glucocorticoids, Glucocorticoid-sparing agents, Tocilizumab, Methotrexate

## Abstract

Die Riesenzellarteriitis (RZA) ist in der Altersgruppe der über 50-Jährigen die häufigste idiopathische systemische Vaskulitis. Die Erkrankung bedarf einer zeitnahen Diagnostik und Therapie, um schwere Komplikationen wie eine Erblindung oder einen Schlaganfall zu vermeiden. Die Rezidivneigung erfordert eine mehrjährige, zum Teil lebenslange Glukokortikoid(GC)-Therapie, was das Risiko GC-induzierter Langzeitnebenwirkungen erhöht. Daher wird bei der Mehrzahl der Patienten eine additive GC-einsparende Therapie empfohlen. Hierzu steht der Anti-IL-6-Rezeptor-Antikörper Tocilizumab in subkutaner Applikation als zugelassene Substanz zur Verfügung, alternativ kann Methotrexat (MTX) eingesetzt werden (off-label).

## Abstract (en)

Giant cell arteritis (GCA) is the most common idiopathic systemic vasculitis in the age group over 50 years. It requires prompt diagnostics and treatment to avoid severe complications, such as visual loss or stroke. The tendency to relapse makes a glucocorticoid (GC) treatment necessary for several years and sometimes lifelong, which increases the risk of GC-induced long-term side effects. Therefore, additive GC-sparing treatment is recommended in the majority of patients. For this purpose, the anti-IL‑6 receptor antibody tocilizumab is available as an approved substance for subcutaneous application; alternatively, methotrexate (MTX) can be used (off-label).

Die Riesenzellarteriitis (RZA) ist in Europa jenseits des 50. Lebensjahrs die häufigste idiopathische Vaskulitis, betrifft überwiegend Frauen und wird bei Beteiligung der oberflächlichen Schläfenarterienäste von neu aufgetretenen, oft temporal lokalisierten starken Kopfschmerzen begleitet. Sie birgt die Gefahr akuter ischämischer Komplikationen mit plötzlicher Erblindung und Schlaganfall oder transitorischer ischämischer Attacke (TIA) und ist daher ein Notfall, der eine rasche Diagnostik und Therapie erfordert. Dieses Review stützt sich im Wesentlichen auf die aktuellen S2k-Leitlinien von Großgefäßvaskulitiden und die S1-Leitlinien der Deutschen Gesellschaft für Neurologie (DGN) zur zerebralen Beteiligung bei systemischen Vaskulitiden.

Nach der revidierten Nomenklatur der Chapel-Hill-Konsensuskonferenz (CHCC) der systemischen Vaskulitiden aus dem Jahr 2012 gehört die Riesenzellarteriitis (RZA) zur Gruppe der Großgefäßvaskulitiden [24]. Die früher häufig synonym verwendete Bezeichnung „Arteriitis temporalis“ (AT) wurde verlassen, weil nicht bei allen RZA-Patienten die Arteria temporalis betroffen ist und andererseits das Gefäß auch bei anderen Vaskulitiden beteiligt sein kann [3].

## Epidemiologie

Die RZA ist eine typische Erkrankung der zweiten Lebenshälfte und tritt so gut wie nie vor dem 50. Lebensjahr auf [40]. Für Deutschland liegt die Inzidenz in der Altersgruppe über 50 Jahre bei 440 Fällen pro 1 Mio. Einwohnern jährlich [20]. In Europa ist die RZA in dieser Altersgruppe die häufigste idiopathische Vaskulitis [40], Frauen sind 2‑ bis 6‑mal häufiger betroffen.

## Ätiologie und Pathophysiologie

Die Ätiologie der RZA ist nach wie vor ungeklärt. Eine aufgrund jahreszeitlicher Schwankungen der Inzidenz diskutierte infektiöse Ursache konnte nicht bewiesen werden. Aufgrund jahreszeitlicher Schwankungen und einer höheren Inzidenz in Ballungsräumen werden auch Umweltfaktoren als potenzielle Trigger angenommen.

Pathophysiologisch gilt die Erkrankung als T‑Zell-abhängige Autoimmunerkrankung, bei der aus noch unklarem Grund aktivierte dendritische Zellen CD4-positive T‑Zellen und Makrophagen in die Gefäßwand rekrutieren, die dann dort eine granulomatöse Entzündung bewirken [47]. Aktivierte T‑Zellen sezernieren verschiedene proinflammatorische Zytokine. Bei der RZA wurden zwei pathogenetisch relevante T‑Helfer-Zellsubtypen identifiziert: die Th1-Zellen (produzieren insbesondere Interferon-γ) und die Th-17-Zellen (produzieren Interleukin[IL]-6 und IL-21). Th-17-Zellen, nicht aber Th1-Zellen, werden durch eine Glukokortikoidbehandlung wirksam unterdrückt.

## Klinisches Bild und neurologische Symptomatik

Bei ca. 80 % der Patienten steht die klinisch gut erkennbare Entzündung der oberflächlichen Schläfenarterienäste (A. temporalis superficialis) im Vordergrund [10]. Leitsymptom dieser kranialen RZA-Form sind subakut und teilweise *akut einsetzende, anhaltende* und als *sehr stark* empfundene, oft *temporal, *manchmal aber auch parietal oder okzipital* lokalisierte Kopfschmerzen*, die unter Einnahme üblicher Analgetika kaum zurückgehen. Obwohl auch der Spannungskopfschmerz häufig ist, und eine Migräne sich atypisch präsentieren kann, sollte im fortgeschrittenen Alter bei Kopfschmerzen stets an sekundäre Kopfschmerzursachen wie die RZA gedacht werden [44]. Bei der klinischen Untersuchung imponieren *Veränderungen der Temporalarterien* in Form von Druckschmerzhaftigkeit, Schwellung, Verhärtung und abgeschwächtem Puls.

Den Kopfschmerzen geht als Zeichen der systemischen Entzündungsreaktion oft eine „*B‑Symptomatik*“ mit allgemeinem Krankheitsgefühl, Fieber, Gewichtsverlust und Nachtschweiß voraus [3, 10].

Pathognomonisch sind zudem Schmerzen oder eine schmerzlose Kiefersperre beim Kauen (Claudicatio masticatorica; ca. 30 % der Fälle; [29]), die gehäuft auch ein Vorbote RZA-assoziierter Sehstörungen zu sein scheinen [15, 40]. Ursache sind entzündlich bedingte Stenosen im Bereich der Äste der A. carotis externa, die zu einer Ischämie der Kaumuskulatur führen. Bei ausgeprägter oder länger dauernder Symptomatik kann auch ein Gewichtsverlust aufgrund schmerzbedingt reduzierter Nahrungsaufnahme resultieren.

*Transiente oder permanente Sehstörungen wie Amaurosis fugax, Diplopie, akuter Visusverlust bis zur ein- oder beidseitigen Erblindung* sind die Warnzeichen einer okulären Beteiligung [15, 40]. Ursache dafür ist meist eine anteriore ischämische Optikusneuropathie (AION), die durch einen entzündungsbedingten Verschluss der posterioren Ziliararterien mit konsekutiver Ischämie des Sehnervenkopfes verursacht wird.

Nach Eintreten eines einseitigen Sehverlustes ist ohne Therapie bei ca. 60 % der Patienten innerhalb weniger Tage auch das andere Auge betroffen [1]. Eine Ischämie der extraokulären Augenmuskeln kann zu Doppelbildern führen, die im Gegensatz zur eingetretenen Erblindung unter Therapie in der Regel reversibel sind [18].

In bis zu 60 % der Fälle zeigen RZA-Patienten zudem Symptome einer *Polymyalgia rheumatica* (PMR), die sich vor allem mit Morgensteifigkeit und proximal betonten Myalgien an Oberarmen und Schultern sowie Schmerzen in der Muskulatur des Beckengürtels und der Oberschenkel präsentiert [11]. Die PMR ist mit ca. 50 % auch das häufigste Symptom eines RZA-Rezidivs. Eine begleitende Antriebslosigkeit und Abgeschlagenheit kann mit einer Depression verwechselt werden.

Zerebrale Ischämien als Folge einer entzündlichen Beteiligung hirnversorgender supraaortaler Gefäße treten bei ca. 3–7 % der Betroffenen auf [15, 40]. Dabei scheinen das vertebrobasiläre Stromgebiet und hier speziell die Abschnitte V2, V3 und proximale V4 der Vertebralarterien häufiger betroffen zu sein [10, 15, 40]. Eine intrakranielle Gefäßbeteiligung ist sehr selten. Im Vergleich mit der Kontrollgruppe fanden sich bei Schlaganfallpatienten signifikant häufiger okuläre ischämische Symptome (63 % vs. 50 %; *p* < 0,001), weniger CRP-Erhöhung und Anämie [8].

Durch den zunehmenden Einsatz moderner Bildgebungsverfahren ist bekannt, dass bei über 50 % der Patienten mit gesicherter RZA auch eine entzündliche* Beteiligung der Aorta und der Arterien der oberen Extremitäten* vorliegt [17]. Am häufigsten betroffen ist die A. axillaris, gefolgt von der distalen A. subclavia und proximalen A. brachialis [40]. Das Beschwerdebild kann je nach Stenosegrad der betroffenen Gefäße von Claudicatio-Symptomen des Arms über Ruheschmerzen und Parästhesien bis hin zur Gangrän von Fingern und der Hand reichen. Eine aortale Beteiligung im Sinne einer Aortitis verläuft klinisch oft stumm, geht jedoch mit einem im Verlauf erhöhten Risiko für thorakale Aneurysmata einher. Diese sind durch das Dissektionsrisiko mit einer 5,1-fach erhöhten Mortalität assoziiert [26]. Einer Auswertung von Daten des französischen Sterberegisters zufolge beruhten die RZA-assoziierten Todesfälle vor allem auf Aortenaneurysma und -dissektion, arterieller Hypertonie, Diabetes mellitus, Infektionen und koronarer Herzerkrankung (KHK; [2]).

## Diagnostik

Bei neu aufgetretener RZA besteht eine erhebliche Erblindungsgefahr, der Anteil permanenter Visusverluste wird in größeren Studien mit ca. 6–37 % angegeben [15, 40]. Die Differenz der Prävalenz kommt vermutlich durch die unterschiedlichen Kollektive und Einschlusskriterien zustande [40]. Ein bleibender Visusverlust tritt in aller Regel vor Einleitung einer Therapie mit Glukokortikoiden (GC) auf, danach nur noch selten [15, 40]. Daher ist ein rasches diagnostisches und therapeutisches Handeln essenziell. Entscheidend ist, dass sich die Behandlung nicht durch die diagnostische Abklärung verzögert, sondern bereits bei bestehendem Verdacht eingeleitet wird.

Mit der Etablierung sog. „Fast-track“-Sprechstunden, in denen bei Verdacht auf eine RZA innerhalb von 24 h eine fachspezifische Abklärung inklusive Ultraschalldiagnostik erfolgt und die Therapie entsprechend früh eingeleitet wird, lässt sich das Risiko einer Erblindung signifikant reduzieren [35].

### Anamnese, klinische Untersuchung und Labor

Neben Anamnese und klinischer Untersuchung gehört eine Blutentnahme zur Basisdiagnostik. Es gibt bisher keinen spezifischen Labormarker für die RZA. Jedoch findet sich bei fast allen Patienten mit histologisch gesicherter Erkrankung zum Zeitpunkt der Diagnose ein Anstieg des C‑reaktiven Proteins (CRP) und/oder der BSG als wichtigsten Laborparametern; eine aktive RZA mit negativem CRP ist extrem selten. Darüber hinaus liegen häufig erhöhte Fibrinogenwerte sowie eine Thrombozytose und Hyperferritinämie vor. Bei längerer Latenz zwischen Beginn der Symptomatik und Vorstellung beim Arzt und Diagnose einer RZA – im Durchschnitt 9 Wochen [38] – kann zudem eine mikrozytäre hypochrome Anämie vorhanden sein, bedingt durch die chronische Entzündung. Geht der Patient jedoch mit akuter Erstmanifestation gleich zum Arzt, kann die Anämie fehlen.

### Farbkodierte Duplexsonographie

Derzeit empfohlene Linearschallköpfe mit >15 MHz verfügen bei einer lateralen und axialen Ortsauflösung von 0,1 mm über eine höhere Auflösung als die MRT und CT. Aufgrund des segmentalen Befalls sollten immer die Temporalarterie (TA) selbst sowie deren parietale und temporale Äste untersucht werden.

Als extrakraniale Gefäße sind am häufigsten die Aa. axillares und subclaviae betroffen, überwiegend bilateral und in 50 % mit Stenosen einhergehend. Daher wird initial immer auch die Sonographie der A. axillaris empfohlen [33, 40].

**Figure 1:**
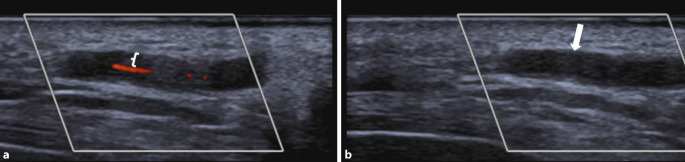

Leitbefund der farbkodierten Duplexsonographie (FKDS) ist das *Halo-Zeichen* [12, 15, 40]. Dabei handelt es sich um einen echoarmen, meist konzentrischen Randsaum um das Lumen der betroffenen Arterie, der in zwei Ebenen sichtbar und nicht komprimierbar ist und durch das entzündliche Infiltrat und das begleitende Ödem verursacht wird ([33, 40]; Abb. 1). Zudem kommt es zum Verlust der dreischichtigen Echodarstellung der betroffenen arteriellen Abschnitte sowie zur Zunahme der Intima-Media-Dicke (IMT).

Bei typischer Symptomatik und positiver FKDS-Bildgebung mit Halo-Zeichen kann auf weitere diagnostische Maßnahmen, auch auf eine Biopsie der oberflächlichen TA, verzichtet werden [12, 31, 40]. Die bereits hohe Sensitivität (>80 %) und Spezifität (>90 %) der FKDS lässt sich durch eine Temporalarterienbiopsie (TAB) kaum steigern [40].

Zu berücksichtigen ist, dass sich Halos der A. temporalis unter Therapie zurückbilden und in 95 % der Fälle innerhalb weniger Tagen bis einiger Monate verschwinden können; die Sensitivität der FKDS kann daher 2 Tage nach GC-Beginn nur noch 50 % betragen [31]. Das unterstreicht die Bedeutung einer zeitnahen Durchführung der Untersuchung.

### Temporalarterienbiopsie und Histologie

Sofern ein klinisch begründeter Verdacht auf eine kranielle RZA besteht und in der Duplexsonographie oder mit einem anderen bildgebenden Verfahren keine Arteriitis zu beweisen ist, empfehlen die S2k-Leitlinien zur histologischen Diagnosesicherung eine in Lokalanästhesie durchgeführte TAB [40].

Da die RZA häufig einen diskontinuierlichen Befall der Gefäße aufweist („skip lesions“), ist die Sensitivität der TAB abhängig von einer ausreichenden Probenlänge des Gefäßes [40]. Die Biopsatlänge sollte daher mindestens 1 cm und idealerweise 1,5 cm betragen. Eine beidseitige TAB erhöht die diagnostische Wahrscheinlichkeit einer RZA nur gering.

Da die Persistenz des entzündlichen Infiltrates nach Beginn der GC-Therapie sinkt, und damit die Sensitivität der TAB nach ≥7 Tagen um ca. ein Drittel reduziert ist [31], sollte die Untersuchung innerhalb weniger Tage nach Beginn der GC-Therapie durchgeführt werden [40]. Jedoch besteht mindestens bis zu 4 Wochen nach Beginn der Behandlung Aussicht auf eine positive Histologie [23, 40].

Nach den Kriterien des American College of Rheumatology (ACR) umfassen die charakteristischen histopathologischen Merkmale der RZA eine Vaskulitis mit prädominant mononukleärer Zellinfiltration oder granulomatöser Entzündung meist mit mehrkernigen Riesenzellen (fusionierte Makrophagen; [22]). Typisch ist eine überschießende Proliferation der Intima, die zur Einengung des Gefäßlumens bis hin zum kompletten Gefäßverschluss führt. Zudem liegt in der Regel eine transmurale Entzündung durch T‑Lymphozyten und Makrophagen mit Verdichtung im Bereich von Lamina elastica interna und Lamina elastica externa (bzw. am Übergang von Media und Adventitia) vor [40]. Allerdings sind nicht immer alle morphologischen Diagnosekriterien erfüllt: Erfahrungsgemäß finden sich bei bis zu 50 % der RZA-Patienten in der TAB keine Riesenzellen [40]. Bei nicht eindeutigen Befunden sollte eine zweite pathologische Meinung eingeholt werden.

Da die RZA die Gefäße häufig nur segmental befällt, ist die Diagnose bei negativer Biopsie nicht ausgeschlossen. So zeigte eine retrospektive Analyse, dass bei 154 Patienten mit negativer Biopsie bei 31 (20 %) eine RZA vorlag [6]. Bei begründetem klinischem Verdacht sollte daher bei negativer Biopsie ein weiteres bildgebendes Verfahren zur Diagnosesicherung eingesetzt werden.

### Fakultative Diagnostik mittels Bildgebung

Zur diagnostischen Sicherung sind bei der RZA verschiedene bildgebende Verfahren geeignet. Etabliert haben sich neben der First-line-Diagnostik mittels der FKDS zusätzlich fakultativ noch die hochauflösende Magnetresonanztomographie (MRT), die Computertomographie (CT) bzw. CT-Angiographie (CTA) sowie die ^18^F‑Fluordeoxyglukose-Positronenemissionstomographie (FDG-PET) mit CT (FDG-PET-CT).

Da keine dieser Methoden eine 100 %ige Sensitivität aufweist, erfordern negative Ergebnisse eine Betrachtung im klinischen Gesamtkontext und die Kombination mehrerer diagnostischer Verfahren ([40]; Tab. 1).

**Table 1:** 

Wenn Klinik eindeutig ist und Sonographiebefund (oder erste Bildgebung; nicht nur Sonographie) passt: keine weitere diagnostische Methode notwendig
Klinik untypisch und Sonographiebefund (1. Bildgebung) passt: zweite diagnostische Methode (2. Untersuchung = Bildgebung oder TAB) erwägen
Klinik typisch und Sonographiebefund nicht eindeutig: 2. Untersuchung = Bildgebung (MRT oder alternativ PET) oder TAB
Klinik und Sonographiebefund negativ: MRT der Temporalarterien und anderer Großgefäße
Sonographiebefund und Biopsie negativ: MRT mit „black blood“; wenn das negativ ist: PET
Bei Axillarisbeteiligung in Sonographie: CT oder MRT zum Ausschluss Großgefäßbeteiligung
PET ist für kranielle Beteiligung nicht geeignet, weil durch Überlagerung durch Glukose-Uptake des Gehirns A. temporalis nicht mit allen Scannern darstellbar ist

#### Hochauflösende Magnetresonanztomographie

**Figure 2:**
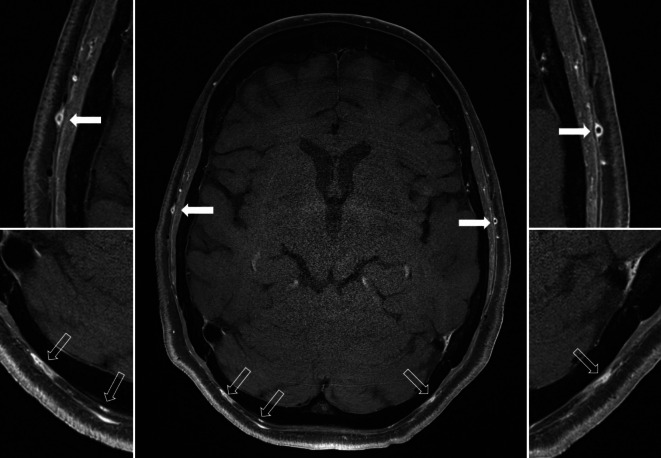

Mit dieser Methode lassen sich mit einer hohen räumlichen Auflösung murale Entzündungszeichen wie Wandverdickung >600 μm und vermehrte Kontrastmittelaufnahme der oberflächlichen TA präzise und untersucherunabhängig darstellen ([5, 28]; Abb. 2). Die Untersuchung ist bei 1,5 und 3 T mit gleicher Auflösung möglich, allerdings kann mit 3 T-Scannern aufgrund der höheren Signalausbeute eine bessere Bildqualität erreicht werden [40].

Dabei ist zu beachten, dass auch die vaskulitischen MRT-Zeichen unter erfolgreicher GC-Therapie sehr schnell schwächer werden und bei Remission vollständig verschwinden [5]. Daher sollte die Bildgebung vorzugsweise innerhalb der ersten 5 Tage nach GC-Therapiebeginn erfolgen.

**Figure 3:**
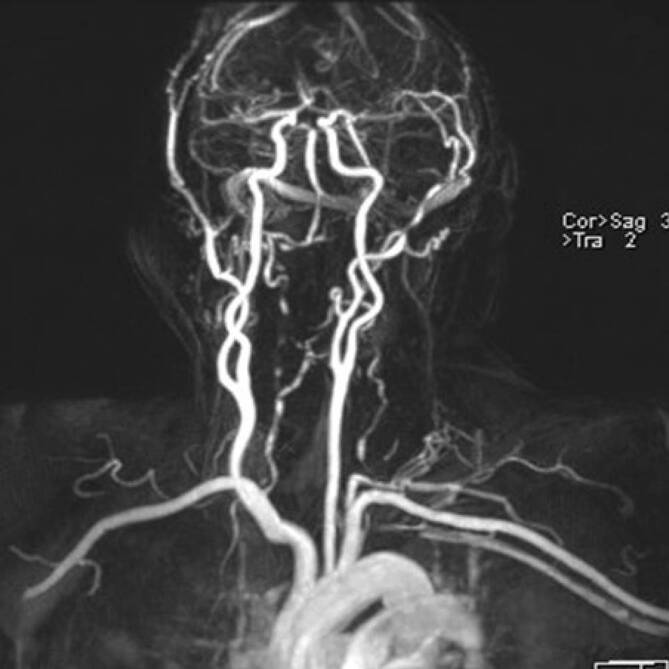

Die MRT der Kopfschwarte kann mit einer MR-Angiographie der Aorta und ihrer Äste kombiniert werden und somit die Beteiligung der extrakraniellen großen Arterien von der Karotisbifurkation bis zu den Oberbaucharterien evaluiert werden [5, 40]. Mit der sog. „Black-blood“-Technik können auch entzündete Segmente der intrakraniellen Arterien beurteilt werden (Abb. 2). Zu berücksichtigen ist dabei, dass eine Kontrastmittelaufnahme nicht spezifisch für entzündliche Veränderungen ist, sondern auch bei nicht entzündlichen Erkrankungen wie Moyamoya, Dissektion und dem reversiblen zerebralen Vasokonstriktionssyndrom (RCVS) zu finden ist [36]. Die MR-Angiographie ist auch geeignet zur Darstellung entzündlicher Gefäßwandveränderungen der A. vertebralis (Abb. 3).

#### ^18^F-Fluordeoxyglukose-Positronenemissionstomographie

Die FDG-PET gilt als das sensitivste Verfahren zur Darstellung auch einer geringen entzündlichen Beteiligung der großen extrakraniellen Gefäße, da selbst eine FDG-Aufnahme im pikomolaren Bereich in der FDG-PET dargestellt werden kann [5, 16].

#### Computertomographie und CT-Angiographie

Auch in der CT und CT-Angiographie (CTA) zeigen sich im Einzelfall Hinweise auf das Vorliegen einer kraniellen RZA mit Beteiligung der Temporalarterien [40]. Vorteile der Methoden sind ihre breite Verfügbarkeit, die schnelle Durchführbarkeit und die standardisierte Datenakquisition und damit geringere Untersucherabhängigkeit [5]. Allerdings setzt die zur Beurteilung einer möglichen muralen Entzündungsaktivität der Aorta und ihrer großen Äste notwendige intravenöse Kontrastmittelgabe eine gute Nierenfunktion voraus [5].

## Differenzialdiagnosen

Eine Reihe von Erkrankungen zeigt überlappende Symptome und Befunde und sollte daher bei der Differenzialdiagnose berücksichtigt werden.

An erster Stelle stehen hier primäre Kopfschmerzen wie chronischer Spannungskopfschmerz, die im Alter zwar seltener als bei jungen Menschen auftreten, aber immer noch häufig sind, sowie Migräne, die bei älteren Menschen häufiger atypisch lokalisiert sein kann (nuchal, temporal; [4]). Auch Kopfschmerzen anderer Ursachen, wie beispielsweise im Rahmen einer Sinusvenenthrombose, parainfektiöser Kopfschmerz und sekundäre Kopfschmerzsyndrome (z. B. Kopfschmerz im Rahmen einer Moyamoya-Angiopathie) müssen abgeklärt werden.

Weitere Differenzialdiagnosen sind Arteriitis der Vasa vasorum der Temporalarterien z. B. im Rahmen einer ANCA-assoziierten Vaskulitis, eine nichtarteriitische AION und eine Endokarditis (erhöhte Entzündungswerte, allgemeine Abgeschlagenheit, ggf. neurologische Ausfälle bei septischen zerebralen Embolien; [3]).

## Verlauf und Prognose

Nach heutiger Einschätzung besteht ein relevantes Risiko für krankheits- und therapiebedingte Komplikationen. So erleidet trotz initial guten Ansprechens auf eine GC-Monotherapie mehr als die Hälfte der Betroffenen innerhalb des ersten Jahres nach Therapiebeginn ein Rezidiv, knapp 80 % in den ersten 5 Jahren [7].

Bei Frauen, Patienten mit systemischen Manifestationen (z. B. Fieber) bei der Erstmanifestation sowie bei einer Steroidtherapie <10 mg Prednisolon/Tag besteht ein erhöhtes Rezidivrisiko [13, 30]. Einige Arbeiten zeigen zudem ein höheres Rezidivrisiko bei Beteiligung der extrakraniellen Gefäße, das sich mit einer aktiven arteriellen Entzündung in der FDG-PET-CT trotz klinischer Remission erklären lässt [16].

## Therapie

**Figure 4:**
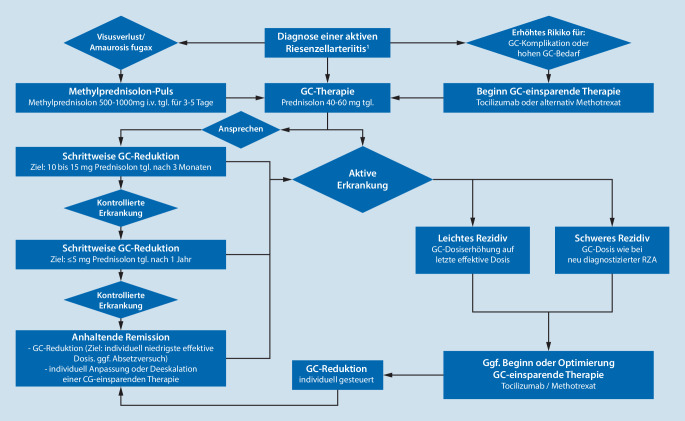

Die Therapieziele bei Patienten mit RZA sind das Erreichen einer Remission und die Prävention akuter ischämischer Komplikationen sowie von Langzeitschäden. Es gibt keine evidenzbasierten Kriterien für die Remission, nach den EULAR-Empfehlungen ist sie definiert als die Abwesenheit aller klinischen Anzeichen und Symptome, Normalisierung von BSG und CRP sowie bei Patienten mit extrakranieller Beteiligung fehlendem Hinweis auf progrediente Gefäßstenosen oder -dilatation [19] (Abb. 4).

### Glukokortikoide

Der begründete Verdacht auf eine RZA und das Vorliegen akuter Visusstörungen ist als Notfall einzustufen und nach Infektausschluss eine sofortige, innerhalb von 24 h eingeleitete präemptive Behandlung mit GC notwendig, um das Risiko einer Erblindung zu minimieren [40]. Die empfohlene Dosis dieser GC-Pulstherapie liegt bei 500–1000 mg Methylprednisolon i.v. täglich über 3 bis 5 Tage.

Auch bei Patienten mit Verdacht auf RZA ohne Visusstörungen sollte die Diagnose noch am Tage der Erstvorstellung oder maximal innerhalb von 2 bis 3 Tagen mittels Bildgebung oder ggf. Biopsie diagnostisch gesichert werden. Ist die Diagnosesicherung nicht umgehend möglich, sollte der Patient schnellstmöglich einem interdisziplinären Team oder Zentrum vorgestellt werden, wo die Entscheidung zur Therapie je nach diagnostischem Befund getroffen wird. Bei diesen Patienten wird eine GC-Dosis von initial 40–60 mg Prednisolonäquivalent täglich empfohlen [40].

Die GC-Wirkung setzt dabei unterschiedlich schnell ein: An den oberflächlichen Temporalarterien ist ein Effekt klinisch bereits sehr rasch innerhalb einer Stunden bis Tagen zu erkennen. An der Aorta können Wandverdickung und Mehrkontrastierung im MRT über viele Monate persistieren, auch in klinischer Remission. Bei über 50 % der RZA-Patienten in klinischer Remission lässt sich im PET-CT in der Aorta oder in aortennahen Gefäßen ein Signal nachweisen [39].

### Sonstige medikamentöse Therapien

Nach früheren Empfehlungen haben alle RZA-Patienten zur Prävention ischämischer Komplikationen primärprophylaktisch Acetylsalicylsäure (ASS) erhalten. Die S2k- und EULAR-Leitlinien sehen dafür heute keine ausreichende Evidenz mehr, sodass keine generelle Indikation für ASS bei RZA-Patienten vorliegt, sondern nur bei gegebener Zusatzindikation (z. B. KHK, signifikante Gefäßstenosen; [19, 40]). Sollte es zu einem Schlaganfall oder einer TIA infolge der RZA gekommen sein, ist eine Sekundärprophylaxe mit ASS 100 mg weiterhin indiziert.

Laut den DGN-Leitlinien lassen retrospektive Kohortenstudien erkennen, dass die Gabe von ASS 100 mg täglich das Risiko kardiovaskulärer Ereignisse bei einer RZA reduzieren könnte [3]. Die Daten sind allerdings nicht eindeutig und kontrollierte Studien zu dieser Fragestellung liegen nicht vor.

Darüber hinaus sollte eine Osteoporoseprophylaxe gemäß den aktuellen Empfehlungen des Dachverbands Osteologie (DVO) eingeleitet werden [3]. Bei GC-Dosen über 20 mg kann in Analogie zu den Empfehlungen bei ANCA-assoziierten Vaskulitiden eine *Pneumocystitis-carinii*-Prophylaxe mit Trimethoprim + Sulfamethoxazol (400 + 80 mg Mo., Mi., Fr. je 2 Tabletten – cave: DANI) erwogen werden.

### GC-einsparende Therapie

#### Tocilizumab

Die bisher einzige in Europa zugelassene GC-sparende Therapie für die RZA ist der Interleukin(IL)-6-Rezeptorblocker Tocilizumab (TCZ; [45]). Die S2k-Leitlinien empfehlen bei gegebener Indikation für eine GC-sparende Therapie nach individueller Abwägung eine CG-einsparende Therapie mit Tocilizumab [40].

Rationale für die Verwendung des monoklonalen Antikörpers ist die pathophysiologische Bedeutung von IL‑6 als zentraler Promotor der chronischen Entzündung und Autoimmunität bei der RZA [46]. Durch die spezifische Bindung des monoklonalen Antikörpers an lösliche und membrangebundene IL-6-Rezeptoren wird die IL-6-vermittelte Entzündungsreaktion verhindert [43]. Tocilizumab erzielt vermutlich seine therapeutischen Effekte unter anderem über eine Modulation dysfunktionaler T‑regulatorischer Zellen (TH17-Zellen).

In der Zulassungsstudie GiACTA wurde die GC-Dosis in den TCZ-Behandlungsarmen innerhalb von 26 Wochen vollständig ausgeschlichen [42]. Mehr als 50 % der Studienteilnehmer blieben unter diesem Prozedere auch nach 52 Wochen in Remission. Daher sollte laut den S2k-Leitlinien unter GC-einsparender Therapie mit TCZ unter entsprechenden Kontrollen eine raschere GC-Reduktion erwogen werden. Tab. 2 zeigt das in der GiACTA-Studie eingesetzte GC-Tapering-Schema (Tab. 2).

**Table 2:** 

Woche	Dosis (mg)
1	60
2	50
3	40
4	35
5	30
6	25
7	20
8	15
9	12,5
11	10
12	9
13	8
14	7
15	6
17	5
19	4
21	3
23	2
25	1
27	0

Angaben in Prednisonäquivalent. Diese schnelle Dosisreduktion bezieht sich nur auf Patienten, die mit Tocilizumab behandelt werden und bei denen eine engmaschige Verlaufskontrolle durch einen versierten Arzt sichergestellt ist. In anderen Fällen sollten auch langsamere Reduktionsschemata zur Anwendung kommen. Jeder Reduktionsschritt sollte nur erfolgen, sofern es keine Anzeichen einer zunehmenden Krankheitsaktivität oder eines refraktären Verlaufes gibt. Zu beachten ist die stark eingeschränkte Aussagekraft des CRP-Wertes unter Tocilizumab

##### Wichtige Voruntersuchungen, Besonderheiten und Labormonitoring.

Vor Beginn einer Therapie mit TCZ sollte trotz fehlender Evidenz für ein gehäuftes Neuauftreten oder eine Aktivierung einer Tuberkulose ein Tbc-Screening durchgeführt und bei positivem Resultat eine prophylaktische Therapie mit Tuberkulostatika durchgeführt oder eine alternative Therapie (z. B. Methotrexat [MTX]) erwogen werden [14, 45]. Zudem erfolgt ein Screening auf Hepatitis B.

Absolute Kontraindikationen einer TCZ-Therapie sind akute Infektionen; bei Patienten mit intestinalen Ulzerationen oder Divertikulitis in der Anamnese sollte TCZ wegen des möglichen Risikos einer gastrointestinalen Perforation mit Vorsicht angewendet werden [45]. Eine Divertikulose ist keine Kontraindikation, sodass eine Koloskopie vor Beginn der Therapie mit TCZ nicht notwendig ist.

Unter Therapie mit TCZ empfiehlt sich eine klinische und laborchemische regelmäßige Überwachung [14]. Zu berücksichtigen ist, dass bei akuten (auch bakteriellen) Entzündungen unter Therapie mit TCZ Akute-Phase-Reaktanten wie CRP nicht oder weniger stark ansteigen und damit das CRP nicht richtungsweisend sein kann [14, 45]. Daher müssen Patienten darüber informiert werden, umgehend ihren Arzt zu kontaktieren, sobald Symptome einer Infektion auftreten, damit eine schnelle Abklärung und Behandlung erfolgen kann.

#### Methotrexat

Es liegen aktuell drei randomisiert-kontrollierte Doppelblindstudien zur Wirksamkeit von MTX als additive immunsuppressive Basistherapie bei RZA vor, deren Ergebnisse uneinheitlich sind [3].

Jover et al. (*n* = 42) beschrieben, dass die Kombination von MTX 10 mg p.o. wöchentlich mit Prednisolon (verglichen mit Placebo + Prednisolon) mit einer Reduktion des Rezidivrisikos und einer niedrigeren kumulativen GC-Dosis assoziiert war [25]. Zwei weitere Studien ergaben keinen Nutzen einer MTX/GC-Kombination im Vergleich mit einer GC-Monotherapie plus Placebo [21, 41], allerdings schränkten die relativ niedrigen eingesetzten MTX-Dosierungen und die kleinen Fallzahlen die Beurteilung ein. Eine Metaanalyse der drei randomisierten kontrollierten Studien, in denen die Wirksamkeit von MTX (7,5–15 mg/Woche) als additive immunsuppressive Basistherapie untersucht wurde, zeigten dann aber eine signifikante Reduktion der Rezidivrate sowie der kumulativen GC-Dosis [32].

Laut den S2k-Leitlinien ist MTX daher eine alternative Option zur GC-einsparenden Therapie, wenn auch in dieser Indikation formal nicht zugelassen [40]. Für andere Immunsuppressiva oder Biologika liegen keine Studiendaten vor, die einen routinemäßigen Einsatz bei der RZA rechtfertigen.

## Verlaufskontrollen

Bei Patienten mit Großgefäßvaskulitiden wie der RZA werden regelmäßig klinische und laborchemische Untersuchungen empfohlen [40]. Dabei sind keine Daten verfügbar, in welchen Intervallen dies idealerweise geschehen soll [34, 40]. Allerdings ist angesichts der hohen Rezidivrate und der möglichen Folgen rezidivbedingter Gefäß- und Organschäden eine engmaschige Kontrolle durch den vaskulitiserfahrenen Facharzt insbesondere zu Erkrankungsbeginn üblich (im ersten Jahr alle ein bis 3 Monate, anschließend alle 3 bis 6 Monate; bei rezidivfreier Remission eventuell jährliche Nachsorge; [40]). Klinische Symptome und Laborwerte (insbesondere CRP) sollten vor allem zu Beginn der Therapie noch engmaschiger durch den Hausarzt überprüft werden. Die Intensität und Art der Kontrollen hängt auch von der gewählten immunsuppressiven Therapie ab. Therapieüberwachungsbögen für Tocilizumab und Methotrexat sind auf der Homepage der DGRh abrufbar (https://dgrh.de/Start/Versorgung/Therapieinformationen/Therapieinformationsbögen.html).

Sofern initial bildgebend ein struktureller Befund an den extrakraniellen Gefäßen (Aneurysma, Stenose) vorlag, kommt eine Langzeitüberwachung der Aorta infrage. Der Zeitpunkt der erneuten Bildgebungskontrolle hängt dabei von der Schwere des initialen Befunds ab. Den Ergebnissen einer aktuellen Arbeit von Kermani et al. zufolge besteht bei initial extrakranieller Gefäßbeteiligung ein erhöhtes Risiko für neue strukturelle Veränderungen, oft auch ohne klinische Symptome einer aktiven Erkrankung [27]. Daher wird bei diesen Patienten eine niederschwellige Kontrolle der Bildgebung empfohlen. In einer Studie aus Schweden [37] kam es im Median nach etwa 3,7 Jahren zu einer Aortendilatation. Eine Studie aus Frankreich zeigte ähnliche Ergebnisse [9]. In Vaskulitiszentren erfolgt zum Teil routinemäßig eine MR-Verlaufskontrolle der Aorta und der supraaortalen Äste initial, nach 6 Monaten und danach jährlich.
